# Impacts of a Home Visiting Program Enhanced with Content on Healthy Birth Spacing

**DOI:** 10.1007/s10995-020-02968-6

**Published:** 2020-07-07

**Authors:** Susan Zief, John Deke, Paul Burkander, Andrew Langan, Subuhi Asheer

**Affiliations:** Mathematica, P.O. Box 2393, Princeton, NJ 08543 USA

**Keywords:** Expectant and parenting youth, Teen parent, Home visiting, Parenting, Contraception, LARC

## Abstract

**Objectives:**

This study sought to determine the impact of Healthy Families Healthy Futures (HFHF) enhanced with Steps to Success (STS). HFHF is a structured home visiting program for teen parents in Houston that focuses on improving parenting skills and preventing child abuse. HFHF enhanced with STS includes content and activities aimed to reduce repeat pregnancies within 24 months after the first child’s birth.

**Methods:**

The study team recruited 248 young mothers for the study, primarily through local health clinics and schools, and then randomly assigned them to either a treatment group that was eligible to participate in HFHF enhanced with STS or to a control group. The control group was not offered any other program through the study. Outcomes were measured by a survey administered 12 months after program intake, in five domains aligned with the program’s logic model: (1) exposure to information related to program content, (2) contraception knowledge, (3) contraception use, (4) enhanced family functioning, and (5) child health and development. To estimate program impacts, we used ordinary least squares regression, controlling for demographics and baseline measures of the outcome variables, if available. We use both frequentist approaches (calculations of statistical significance) and Bayesian posterior probabilities to interpret the findings.

**Results:**

HFHF enhanced with STS significantly (*p* < .05) impacted exposure to information on parenting and birth control, with effects of 20.8 and 15.4 percentage points, respectively. Using Bayesian posterior probabilities, there is an 85% chance that the program had a favorable effect on these outcomes. We also calculate a probability of 77% that the program had a favorable impact on long-acting reversible contraceptive (LARC) use, but a probability of 89% that the program reduced knowledge of birth control pills; these two results were not statistically significant (*p* = .17 and .10, respectively).

**Conclusions for Practice:**

These findings are primarily favorable and consistent with the program content and goals. Smaller than anticipated sample sizes due to recruitment challenges increased the chances for random error to affect the ability to detect statistically significant differences on many of our other outcomes; Bayesian posterior probabilities can therefore aid in interpreting the impact estimates. More research of this promising model is warranted.

**Electronic supplementary material:**

The online version of this article (10.1007/s10995-020-02968-6) contains supplementary material, which is available to authorized users.

## Significance

What is already known on this subject: Home visiting programs for families have demonstrated evidence of effectiveness on outcomes related to maternal and child health, parenting, child maltreatment, and domestic violence (Sama-Miller et al. 2017). However, these programs do not have an explicit focus on the highly vulnerable teen parents and their young children, such as those funded by the Pregnancy Assistance Fund (PAF). Little is known about effective models for the families supported by the PAF program (Person et al. 2018) and in particular on approaches to reduce chances of a repeat pregnancy in the teenage years. Few studies of programs for teen parents have measured impacts on healthy birth spacing (Harding et al. 2020).

What this study adds: This paper examines the impacts of a home visiting program enhanced with information on healthy birth spacing that is specifically designed for young and vulnerable teen mothers and their partners. Study findings provide much needed evidence on effective approaches for improved outcomes for teen parents and their children by mediating the risk of a rapid repeat pregnancy.

## Objectives

Teen pregnancy and birth rates in the United States declined significantly between 1991 and 2016, from about 62 per 1000 females to about 20 per 1000 (Martin et al. 2017). Yet the repeat birth rate among teens—approximately one in six teen births—has not experienced such declines (Dee et al. 2017). A rapid repeat birth during adolescence increases the risk of poorer outcomes for both the mother and children. Teen mothers who experience rapid repeat pregnancies (within 18 months of the prior birth) are at significantly greater risk of having a stillbirth or preterm birth than are teen mothers who delay subsequent childbearing (Conde-Agudelo et al. 2006). They are also less likely to stay in or complete high school, to work or maintain economic self-sufficiency, or to have children who exhibit school readiness when older as compared with other teen mothers (Klerman 2004).

In response to the needs of teen parents in Houston, Texas, the Houston Health Department (HHD) developed Healthy Families Healthy Futures (HFHF), a home visiting program to serve teen mothers. HHD has been implementing HFHF for more than a decade. Traditionally, this program has followed a home visiting format that is modeled after Healthy Families America (HFA), which focuses on preventing child abuse by building parenting skills.

In 2015, to address growing concerns about rapid repeat pregnancies among teens, HHD was looking for ways to modify the program to incorporate components that more directly address contraception and healthy birth spacing, knowledge the HHD found particularly lacking in their teen clients. HHD decided to enhance HFHF with Steps to Success (STS), a 2-year curriculum developed by Healthy Families San Angelo that is intended to be combined with a home visiting program that focuses on the teen mother and her male partner (father of the baby or significant other). STS was designed to address rapid repeat births by providing contraception knowledge, discussing life planning in home visits, and involving the male partner in contraceptive decision making. To deliver STS with fidelity, both program administrators and staff delivering the program must have completed comprehensive training in a standard evidenced-based home visiting intervention such as HFA and be familiar with the specific strategies, approaches, and required benchmarks associated with a high quality, evidenced-informed home visiting program.

**Fig. 1:**
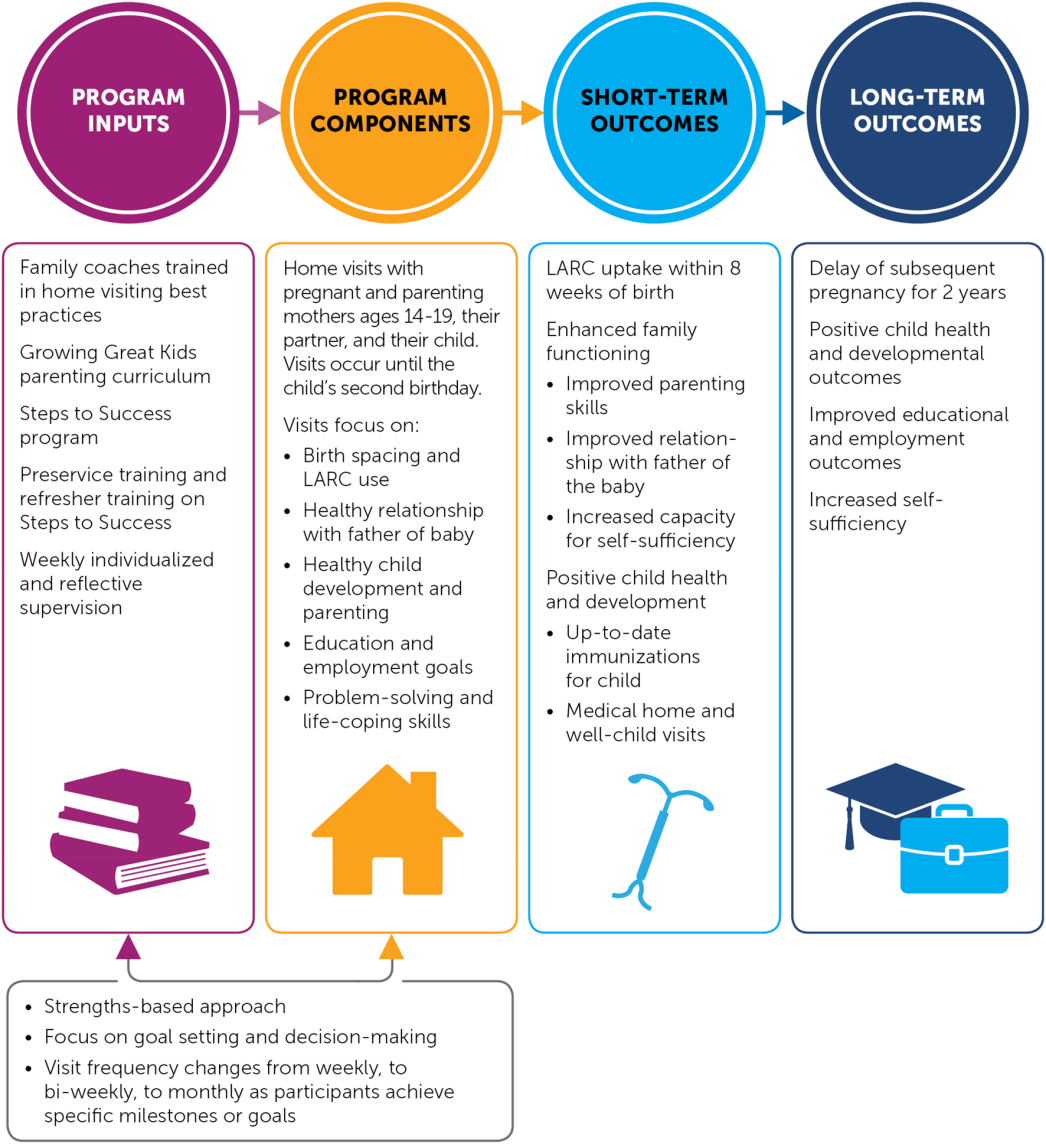
The evaluated Healthy Families Healthy Futures program combines aspects of Healthy Families America, an evidence-based home visiting program, which focuses on building parenting skills and preventing child abuse, and Steps to Success, which addresses contraception and healthy birth spacing

This modified version of Houston’s HFHF (Fig. 1), which integrates the STS components, is the subject of this evaluation and is referred to as HFHF for the remainder of the paper. The program relies on a strengths-based approach to engage and serve teen mothers. Home visits are designed to focus on each young mother’s talents, capacities, and competencies; her family; and her peers to help her enhance connections, remove barriers, set goals, and develop decision-making skills. The program is designed to last for 2 years or until the focal child turns age two. Teen mothers can enter the program when they are either pregnant or parenting a child younger than 3 months. Visit frequency is intended to range from once per week to once per month, depending on the length of time in the program, the client’s needs, and the accomplishment of program milestones. Family coaches, who primarily have backgrounds in social work, are trained to enhance their more traditional HFHF home visiting program with STS content. The family coaches meet with participating pregnant and parenting females, their male partner, and the child to discuss birth spacing, contraception choices, healthy parental relationships, healthy development of the child, parental education and employment goals, and problem-solving and life-coping skills. In the short term (within 1 year of program enrollment), the program seeks to increase the use of long-acting reversible contraception (LARC) by providing education on efficacy, availability, and cost; enhance family functioning, including improving father involvement; and meet the baby’s child development needs. In the long term (by the end of the 2-year program), HFHF aims to delay subsequent pregnancies, ensure positive child development, and increase parents’ self-sufficiency.

This paper examines the impacts of HFHF while also exploring program implementation. Specifically, the paper addresses the successes and challenges of program implementation in its first year, the impacts of the program on the receipt of program components, and the impacts of the program on outcomes that are hypothesized to improve within 1 year of program exposure.

## Methods

To be eligible for the study, a pregnant or parenting teen had to be a first-time mother, pregnant or parenting a baby under the age of 90 days, speak English or Spanish, be age 14–19, and have not been enrolled in HFHF in the past 6 months. Eligible young mothers were referred to HHD by schools; Special Supplemental Nutrition Program for Women, Infants, and Children (WIC) clinics; and other community-based organizations. HHD screened each possible new client for study eligibility, most often over the phone, and then passed the referral to the study team, who conducted a second, in-person eligibility screen. The study team then collected consent for the study, administered the baseline survey, and used a study-developed website to conduct random assignment. Random assignment was conducted in two separate blocks for young mothers who (1) were pregnant or (2) were parenting (and not pregnant) at the time of random assignment. Randomly assigning young mothers within blocks enabled us to ensure the treatment and control groups were balanced with respect to pregnancy status. Participating pregnant and newly parenting teen mothers had an equal chance of being placed in the treatment and control groups. Young mothers were randomly assigned to (1) a treatment group that could receive HFHF or (2) a control group that would not. Both research groups were given information on Project Milestone, a referral program for pregnant and parenting teens offered through the WIC clinics.^1^ The intended sample size for the analysis was 600 participants, but shifting priorities within HHD led to an early end of the intake for the evaluation. Between June 2015 and October 2016, 248 young mothers were enrolled in the study and randomly assigned (Table 1).

**Table 1:** Sample size by strata and treatment status

Stratum	Number of participants assigned to HFHF	Number of participants assigned to the control group	Total	Percent of total
Pregnant at random assignment	96	96	192	77.4
Not pregnant at random assignment	30	26	56	22.6
Total	126	122	248	100

*Source* Baseline survey

This paper draws on survey data from our sample of teen mothers at two points: (1) a baseline survey administered upon enrolling in the study and immediately before random assignment and (2) a 1-year follow-up survey, administered about 12 months after enrollment, when it is hypothesized that short-term outcomes could have been achieved.^2^ We developed the survey primarily from other federal surveys that were previously administered to nearly 6000 young mothers. For this current and prior federal evaluations, the items were pretested with members of the target population, including cognitive interviews. We have provided a list of all measures used for impact estimation and their sources in supplementary material. The survey procedures yielded high survey response rates. All 248 teen mothers in the study sample completed a hard copy baseline survey as part of the study enrollment process conducted by the study team. In total, 223 teen mothers completed the follow-up survey (administered as a web or phone survey, at the preference of the respondent) for a total response rate of 90%. We used data from each of these two surveys to estimate program impacts on the expected short-term outcomes.

A separate implementation study examined actual program implementation against the intended plans for HFHF with STS. The study team collected administrative data on service receipt through a service log completed by the family coaches after home visit. The family coaches recorded the visit date and length; whether the visit was with the mother of the baby, the father of the baby, or both (family coaches were encouraged to meet with the father of the baby if mother and father would not or could not meet together); whether the home visit included a discussion of parent education, resources and/or referrals, contraception, and a long-term reproductive life plan (all components of a home visiting program with STS); whether the teen mother was on contraception if post-partum; and if so, what type. These data were collected from the beginning of the study until the HHD ended the study enrollment in October 2016.

During a three-day site visit in fall 2016, we conducted a staff survey and interviews with four of the five staff available at the time of visit, observed four home visits for content covered and interactions between the young mother and her case manager, and extracted data from 19 case files chosen at random. The interviews were designed to better understand more about the respondent’s background and the HHD; the intended design of the program; staff hiring, training, supervision, monitoring, and support; adequacy of resources for implementation; staff perception of the program; staff experiences delivering the program, including successes and challenges; and the availability of other programming through the HHD or elsewhere in the communities. The study team reviewed the case files for information on the teen mother’s background, program delivery and adherence to the program model, teen mother experience and engagement, challenges faced by the teen mothers and strategies used to support them, and overall challenges and successes of delivering the program. In winter 2017, the study team held in-person interviews with 29 HFHF teen mothers about their experiences in the program.^3^

All data were collected in accord with prevailing ethical principles and reviewed by an Institutional Review Board.

The baseline characteristics of our sample of teen mothers by treatment group are reported in Table 2. Some statistically significant differences were found between groups. Members of the treatment group were less likely to have ever been exposed to information on relationships, education services, and methods of birth control. An F-test for whether the characteristics reported in Table 2 jointly predict treatment status rejects the null hypothesis (i.e., that they do not predict treatment) with a p-value of 0.004. Therefore, in our impact analyses, we include regression controls to account the possible influence of baseline characteristics.

**Table 2:** Healthy families healthy futures sample characteristics at program entry (percent, unless otherwise noted)

Characteristic	Treatment mean	Control mean	Difference	Effect size difference	p-Value	Sample size
Age at baseline (years)	17.40	17.34	0.06	0.04	0.743	247
Race and ethnicity					0.260a	
Hispanic	65.07	61.49	3.58	0.07		248
Black non-hispanic	27.30	35.51	− 8.20	− 0.18		242
Other race, non-hispanic	4.96	2.48	2.48	0.13		242
Enrolled in school at random assignment	63.76	64.41	− 0.65	− 0.01	0.916	245
Highest grade completed					0.342a	
8th grade or below	13.60	19.01	− 5.41	− 0.15		246
9th grade	26.34	17.41	8.93*	0.22		246
10th grade	18.39	20.67	− 2.27	− 0.06		246
11th grade	21.66	26.38	− 4.72	− 0.11		246
12th grade	20.00	16.53	3.47	0.09		246
Ever repeated a grade	20.15	25.01	− 4.87	− 0.12	0.367	244
Ever suspended or expelled	50.00	53.28	− 3.29	− 0.07	0.607	248
In previous 12 months, exposed to information about						
Relationships	6.50	15.71	− 9.21**	− 0.29	0.022	244
Parenting	32.20	35.30	− 3.10	− 0.07	0.608	246
Child health care	38.88	44.41	− 5.53	− 0.11	0.376	245
Education related services	28.08	39.32	− 11.25*	− 0.24	0.063	244
Career counseling or job training	11.30	15.69	− 4.40	− 0.13	0.316	245
Methods of birth control	44.87	64.31	− 19.43***	− 0.39	0.001	248
Percent correct on assessments of knowledge about						
Condoms	50.78	47.39	3.38	0.14	0.257	248
Birth control pills	35.21	37.98	− 2.78	− 0.10	0.452	248
IUDs	20.69	21.74	− 1.05	− 0.04	0.726	248
Other methods	24.10	23.31	0.78	0.03	0.791	248
Use of a LARC first time having sexual intercourse	0.85	0.87	− 0.02	0.00	0.988	233
Unprotected sex first time having sexual intercourse	55.44	58.52	− 3.08	− 0.06	0.626	244
Would be upset if pregnant again in next 2 years	3.98	9.07	− 5.08	− 0.21	0.108	246

Sample sizes differ across characteristics due to missing data. Sample means are regression adjusted and differences are estimated using a regression of the baseline characteristic on the treatment indicator and random assignment strata indicator variables, with standard errors adjusted to account for heteroskedasticity. Effect sizes are calculated using Hedges’ g statistic. An F-test of the null hypothesis that all baseline characteristics jointly predict treatment status has a p-value of .004. *Source*: Baseline survey

^*^Significantly different from zero at the .10 level, two-tailed test

^**^Significantly different from zero at the .05 level, two-tailed test

^***^Significantly different from zero at the .01 level, two-tailed test

^a^Baseline differences of these mutually exclusive variables was assessed using an F-test to determine whether baseline characteristics jointly predict treatment status in a regression that also controlled for stratum. Reported p-values are from this test

### Outcome Selection

To provide a comprehensive assessment of HFHF after 1 year of program enrollment, we assessed impacts on a range of outcomes that align with two stages of the logic model: (1) program components and (2) short-term outcomes (see Fig. 1). Because outcomes are drawn from the follow-up survey administered approximately 1 year after study enrollment, our analysis focused on exposure to program components and short-term outcomes that are hypothesized to be attainable within 1 year of program provision. We did not assess the effectiveness of the program on outcomes that are expected to be achievable or measurable only after 2 years in the program. For example, the program’s long-term goals include a reduction in repeat births within a 2-year period, and we did not assess an impact on that outcome. Also, given that 77% of the sample of teen mothers were pregnant at the time of study enrollment (see Table 1), we would not expect the program to have a short-term effect on a subsequent pregnancy or birth 12 months later.

We selected outcomes in two domains that are related to the expected program components: (1) exposure to information related to program content (six measures) and (2) knowledge of contraception (four measures). The six selected measures of exposure to information reflect the specific, expected content of the visits, including whether the respondent ever attended an individual discussion or group class or session on relationships, parenting, child health care, education, career, or birth control.

To delay a subsequent pregnancy, HFHF seeks to improve participants’ knowledge of contraception methods and access through discussions with clients and their partners, focusing on LARC use in particular. Knowledge of contraception was measured using four multi-item scales with binary response options: knowledge of condoms (six items); knowledge of birth control pills (five items); knowledge of intrauterine devices (IUDs; six items); and knowledge of other hormonal and LARC methods (such as implants, five items). The measures were primarily taken from the Guttmacher Institute Survey of Young Adults (The Fog Zone).^4^

The STS curriculum content added to the original HFHF program was primarily intended to address HHD’s concerns over rapid repeat births among its clients. Within 1 year of program provision, HFHF intended to support clients in reducing sexual risk behaviors that could result in rapid repeat births. Specifically, the program supported clients in making effective birth control decisions with their partners, particularly by providing education on LARC effectiveness and availability. We selected two measures in the domain of use of contraception to reduce sexual risk behaviors: (1) LARC use (defined as either an IUD or an implant) and (2) unprotected sexual intercourse.

In the short term, the program is also intended to affect outcomes that are consistent with a more traditional home visiting program, such as enhanced family functioning through improved parenting skills, the mother’s relationship with the father of the baby, and the mother’s increased capacity for self-sufficiency. We selected five measures in this domain: one on the mother’s relationship with the child; one on the father’s relationship with the child; two on the parents’ co-parenting relationship; and one on the mother’s capacity for self-sufficiency, as evidenced by her attitudes and beliefs toward goal setting, problem solving, and future orientation.

Finally, the HFHF program focuses on supporting the mother to make healthy decisions related to child health and development. We selected two measures in the domain of health and development: (1) number of well-child visits and (2) whether mother has secured health insurance for the baby.

In the short term (within 12-months) of program enrollment, the program was not expected to so quickly make a difference in the repeat pregnancy rates given that nearly half of the sample was pregnant upon intake and it takes time to provide information on and access to contraceptives. Therefore, the study team decided not to measure the impact of a repeat pregnancy 12-months after program enrollment even though the question was asked on the survey. This a priori decision is reflected in our plans registered at *ClinicalTrials.gov*.

### Analytic Methods for Impact Analysis

We estimated the impact of HFHF on each outcome measure using the following equation:

1 $${\text{y}}\_{\text{i}} = \upalpha + \uptau {\text{T}}\_{\text{i}} + {\text{X}}\_{\text{i }}\upbeta + \upepsilon\_{\text{i}},$$

where y_i is the outcome for individual i, T_i is an indicator equal to one for teen mothers assigned to the treatment group and zero for those assigned to the control group, X_i is a vector of individual-level covariates, and $\upepsilon\_{\text{i}},$ is an individual-level error term. To account for baseline differences between the treatment and control groups, and to increase the precision of estimated treatment effects, all main analyses control for race; age at sampling; highest grade completed; an indicator for enrollment in school at random assignment; and all available baseline measures of exposure to information, knowledge of birth control methods, and short-term outcomes. The vector X_i also includes an indicator for pregnancy status at random assignment to account for the stratified random assignment. The estimated parameter τ is the average treatment effect of assignment to HFHF. For each outcome, inference is based on standard errors made robust to heteroskedasticity (White 1980).

The chance of observing a false positive increases with the number of outcomes examined. To account for this, within each outcome domain described above, we adjusted the p-values of every test in order to control the familywise error rate at 5%. The statistical procedure we used to adjust *p-*values is based on the multivariate t-distribution and takes into account correlations among test statistics (Hothorn et al. 2008) that we expect, given the likely correlation of our outcome measures within each domain.

Rather than excluding teen mothers with missing baseline data, our main analysis uses a dummy variable adjustment to address missing baseline data (Puma et al. 2009). Specifically, we impute missing data to a constant and include an indicator variable for each baseline variable that has any missing data. This indicator variable is equal to one for teen mothers whose baseline data were missing before imputation and zero for those whose baseline data were not missing before imputation. Young mothers are only excluded from each main analysis for which they are missing the outcome; we include all teen mothers in the treatment group regardless of their level of participation in the program.

To test the robustness of these results, we re-ran all our program impact analyses without controls for baseline covariates. We also estimated the impact of actually receiving the HFHF intervention using a two-stage least squares framework. In this approach, we used treatment status as an instrument for the likelihood that an individual attended at least one HFHF visit.

### Bayesian Interpretation of Estimates

Because of the widespread misinterpretation of *p*-values and statistical significance (Wasserstein and Lazar 2016; Greenland et al. 2016; Wasserstein et al. 2019; Amrhein et al. 2019), we also report a Bayesian posterior probability—the probability that the true effect of HFHF on each outcome was favorable or unfavorable (meaning, an improvement or decrease in outcomes greater than zero), given our findings. To calculate this probability, we use a standard textbook (for example, Gelman et al. 2013) formula based on Bayes rule (Bayes 1763/1958) to combine two sources of information: (1) the standard error of our impact estimate and (2) how common it is for generally similar interventions to have effects (Bayesian statisticians call this the *prior distribution*).^5^ Both sources of information can help us assess the likelihood that our impact estimate represents an effect of HFHF. All else equal, a smaller standard error implies that the impact estimate is more likely to be close to the true effect. Meanwhile, impact estimates from our study that are similar to the prior evidence are judged more likely to be correct. Bayes rule allows us to combine these two sources into an overall assessment of the likely effect of HFHF.

To develop a prior distribution, we conducted a meta-analysis of all findings on similar outcomes from studies rated moderate or high quality from the Home Visiting Evidence of Effectiveness (HomVEE) review. We chose HomVEE because it is a large, rigorous, systematic review of interventions serving a disadvantaged population of new parents and because HomVEE study descriptions provide enough information to support the analysis.^6^ Our meta-analysis revealed the following information about the prior distribution: slightly more than half of intervention effects are favorable, but large effects are unusual (fewer than 20% of effects are larger than 0.10 standard deviation).^7^ We use a textbook formula (for example, Gelman et al. 2013) to calculate the probability of a favorable effect given our impact estimates and the prior distribution, under the assumption that both our impact estimate and the prior distribution are Gaussian (normal).

## Results

### Program Implementation

It took HHD staff nearly a year after study enrollment began to put systems and staff in place to deliver the program as intended.^8^ When the study began, the three existing family coaches, who had between 3 and 10 years’ experience conducting home visits with teen parents, initially struggled to understand and integrate the STS content because they did not have the expected training from HFA, or any other evidence-based home visiting program, and did not received strong, internal supervision.^9^ By fall 2015, approximately 4 months after study activities began, HHD hired two additional family coaches to join the existing three coaches to meet the growing demand of cases. These two new coaches were familiar with working with teen mothers but had no home visiting formal home visiting experience or training. All five coaches struggled to connect with young mothers randomized to HFHF after random assignment; only 60% of the young mothers randomized to HFHF had more than five visits within their first year of enrollment. Using service log data that the HHD provided after each home visit, we identified 50 teen mothers who received any programming for the 12 months following their enrollment into the study and randomization to HFHF. Among these teen mothers, the service log data demonstrate that they received, on average, 14 visits in their first year after enrollment and that, on average, 3 of these visits included the father of the baby or her significant other. By comparison, mothers in San Angelo, Texas, where the program was implemented and evaluated under the supervision of the developer, received 20 visits on average in their first year (Kisker et al. 2016).

In response, the developer offered additional trainings and supervision to support staff in delivering an evidence-based home visiting program with STS content. Despite early challenges, staff were able to provide content on contraception, birth spacing, parenting, and child development to most HFHF young mothers and also involve the fathers. For example, among the 111 teen mothers who received at least one visit over the course of the study, coaches discussed contraceptive choices with more than three-quarters of the teen mothers at least once and did so in nearly half of all visits recorded in the service log. Staff reports of birth control and LARC uptake among their clients, although declining in the beginning, showed a marked improvement. According to service log data, the family coaches tracked declines in their clients’ (the teen mothers’) use of birth control and LARCs during their early months of visits. The home visitor reported rate of postnatal mothers using any form of birth control climbed to 82% in the final service log received, with nearly 50% reporting using a LARC at that time.

The staff also developed supportive relationships with their clients. Youth reported that the staff were the primary source of information about effective methods of birth control, sometimes supplementing and correcting information they received from the internet, family, and friends. Youth engagement and retention also improved over time, and participating young mothers described a strong relationship with staff who were like a surrogate family member or a close friend.

### Program Impacts

HFHF had a significant and positive effect on teen mothers’ exposure to information on parenting and methods of birth control. After 1 year of access to HFHF, a much larger fraction of the sample of teen mothers reported having been exposed to information on parenting (Table 3). Among the sample of teen mothers, 66% reported having received information on parenting, compared to 45% in the control group. Based on this difference and the distribution of evidence from HomVEE, we calculate an 89% probability that this estimate reflects a true difference between the treatment and control groups. One year of access to HFHF appears to have increased the proportion of teen mothers reporting exposure to information on methods of birth control: about 83% of the treatment group reported receiving information on methods of birth control, compared to about 67% of the control group, and we calculate an 86% probability that this difference reflects a true effect of HFHF. We find no other significant impacts on measures of program components.

**Table 3:** Impacts on program components (percent, unless otherwise noted)

Program component	Treatment mean	Control mean	Impact	p-Value	Adjusted p-value	Sample size
In previous 12 months, exposed to information on						
Relationships	18.16	20.82	− 2.66	0.680	0.997	221
Parenting	65.58	44.76	20.81**	0.010	0.025	220
Child health care	56.75	52.66	4.10	0.614	0.992	221
Education related services	29.37	28.80	0.57	0.930	1.000	220
Career Counseling or job training	18.94	22.03	− 3.09	0.624	0.993	220
Methods of birth control	82.71	67.31	15.41*	0.022	0.062	217
Percent correct on assessments of knowledge of contraception						
Condoms	58.84	61.08	− 2.24	0.470	0.867	220
Birth control pills	46.22	55.08	− 8.86	0.049	0.103	220
IUDs	35.33	34.88	0.45	0.904	1.000	220
Other methods	37.06	36.70	0.36	0.923	1.000	220

Treatment and control group means are regression adjusted. Impacts on binary outcomes are estimated using the linear probability model, with standard errors adjusted to account for heteroskedasticity. All regressions include an indicator for parental status at baseline, indicators for race and ethnicity, educational enrollment, age at random assignment, and all available baseline measures of outcome variables. All p-values are based on a two-sided test, and adjusted p-values control for the familywise error rate using the method in Hothorn et al. (2008). Sample sizes differ across outcomes due to missing outcome data. *Source*: Baseline survey and 12 month follow-up survey

^*^Significantly different from zero at the .10 level after adjusting for multiple comparisons, two-tailed test

^**^Significantly different from zero at the .05 level after adjusting for multiple comparisons, two-tailed test

^***^Significantly different from zero at the .01 level after adjusting for multiple comparisons, two-tailed test

One year of access to HFHF also appears to have decreased teen mothers’ knowledge about birth control pills, relative to the control group, although this difference is not statistically significant. Although both the treatment and control groups were more knowledgeable about birth control pills 1 year after random assignment than they were at random assignment, the control group showed a greater improvement in knowledge of birth control pills. On average, the treatment group correctly answered about 46% of questions about birth control pills, compared to about 55% correctly answered by control group members. We calculate an 89% probability that HFHF had an unfavorable effect on knowledge about birth control pills, given this estimate.^10^

HFHF showed a 11 percentage point increase in LARC use among the treatment group—39% of the treatment group reported using a LARC, compared to 28% of the control group—but the difference was not statistically significant (Table 4). We calculate a 77% probability that this difference reflects a true effect of HFHF. HFHF did not appear to affect any other short-term outcomes.^11^

**Table 4:** Impacts on short-term outcomes (percent, unless otherwise noted)

Program component	Treatment mean	Control mean	Impact	p-Value	Adjusted p-value	Sample size
Contraception use in previous 12 months						
Use of a LARC	38.80	27.71	11.09	0.133	0.171	219
Unprotected sex	23.57	26.92	− 3.36	0.650	0.837	218
Respondent intends to wait two or more years before having next child	90.07	93.36	− 3.29	0.477	a	216
Frequency of parental engagement in last month—scale from 0 (never) to 3 (every or almost every day)						
Mother’s engagement with child	2.48	2.47	0.01	0.907	1.000	217
Father’s engagement with child	1.48	1.54	− 0.07	0.700	0.991	211
Quality of co-parenting relationship—scale from 1 to 5 with higher values representing stronger co-parenting	3.72	3.82	− 0.09	0.479	0.898	216
Father pays half or more of child care costs	67.00	70.09	− 3.08	0.666	0.985	216
Capacity for self-sufficiency—scale from 1 to 4 with higher values representing greater self sufficiency	2.15	2.13	0.02	0.730	0.995	221
Child health and development						
Number of well visits	6.21	6.64	− 0.43	0.442	0.623	200
Has health insurance for child	95.77	93.05	2.72	0.390	0.561	216

Treatment and control group means are regression adjusted. Impacts on binary outcomes are estimated using the linear probability model, with standard errors adjusted to account for heteroskedasticity. All regressions include an indicator for parental status at baseline, indicators for race and ethnicity, educational enrollment, age at random assignment, and all available baseline measures of outcome variables. All p-values are based on a two-sided test, and are adjusted to control for the familywise error rate using the method in Hothorn et al. (2008). Sample sizes differ across outcomes due to missing outcome data. *Source*: Baseline survey and 12 month follow-up survey

^*^Significantly different from zero at the .10 level after adjusting for multiple comparisons, two-tailed test

^**^Significantly different from zero at the .05 level after adjusting for multiple comparisons, two-tailed test

^***^Significantly different from zero at the .01 level after adjusting for multiple comparisons, two-tailed test

^a^In this table, p-values are adjusted for multiple comparisons within outcome domains. Birth spacing intention is the only outcome in its domain, so the adjusted p-value is excluded here

All results were robust to the sensitivity tests described above. Results of the sensitivity tests are included in an Appendix (see Tables 5, 6, 7, and 8).

## Conclusions for Practice

The impact analysis results suggest that HFHF very likely increased teen mothers’ exposure to information on parenting and methods of birth control while decreasing their knowledge of birth control pills relative to what it would have been in the absence of the program. Although HFHF intended to improve teen mothers’ knowledge about contraception, the program’s focus on LARC use might have come at the expense of providing information about birth control pills. The proportion of the treatment group reporting LARC use was 11 percentage points higher than in the control group, and we calculate a 77% probability that HFHF truly had a favorable effect (although this difference was not statistically significant). We find no other statistically significant impacts.

These results are primarily favorable and consistent with the program content, goals, and recent evidence on STS (Rotz and Wood 2018). In a prior study on STS conducted at the site of the developer, after 1 year in the program, study participants were more likely than mothers enrolled in the traditional home visiting program to report using LARCs, and there was also some evidence that the program reduced the prevalence of unprotected sex. In our study, smaller than anticipated sample sizes due to HHD’s earlier than anticipated withdrawal from the study increased the chances for random error to affect the ability to detect statistically significant differences on many of our measures, such as LARC use.

Our results are encouraging considering the challenges staff experienced integrating STS into their existing home visiting program over the first year. In many ways, the challenges and successes that HHD experienced rolling out HFHF with STS were not atypical of a first-year replication of an intensive program in any setting, let alone a large, bureaucratic city agency (Bumbarger and Perkins 2008). The similarities of the impacts to the results of the developer’s optimal implementation of the program (Rotz and Wood 2018) suggest that HFHF program impacts could have been even stronger if implementation had been more successful within the first year.

We conclude that more research of this promising intervention model is warranted. An earlier review reports that LARC use is associated with decreased repeat pregnancies (Baldwin ande Edelman 2013). Broader implementation of this intervention could then be considered if these positive findings can be replicated in a larger experimental study that provides the opportunity to examine longer term impacts on healthy birth spacing.

### Electronic supplementary material

Below is the link to the electronic supplementary material.

Supplementary file1 (DOCX 32 kb)

## Appendix

See Tables 5, 6, 7, and 8.

**Table 5:** Impacts on program components, excluding controls for baseline characteristics but including controls for randomization strata (percent, unless otherwise noted)

Program component	Treatment mean	Control mean	Impact	p-Value	Adjusted p-value	Sample size
In previous 12 months, exposed to information on						
Relationships	20.33	18.54	1.80	0.737	1.000	221
Parenting	64.67	45.73	18.94	0.005	0.027	220
Child health care	53.93	55.61	− 1.68	0.803	1.000	221
Education related services	27.69	30.55	− 2.86	0.643	0.997	220
Career Counseling or job training	17.05	23.98	− 6.93	0.208	0.730	220
Methods of birth control	80.04	70.05	9.99	0.092	0.416	217
Percent correct on assessments of knowledge of contraception						
Condoms	59.58	60.31	− 0.73	0.826	0.999	220
Birth control pills	47.98	53.26	− 5.28	0.183	0.504	220
IUDs	36.70	33.46	3.24	0.357	0.790	220
Other methods	37.02	36.75	0.27	0.939	1.000	220

Treatment and control group means are regression adjusted. Impacts on binary outcomes are estimated using the linear probability model, with standard errors adjusted to account for heteroskedasticity. Regressions control only for treatment status and randomization stratum. All p-values are based on a two-sided test, and adjusted p-values control for the familywise error rate using the method in Hothorn et al. (2008). Sample sizes differ across outcomes due to missing outcome data. Source: Baseline survey and 12 month follow-up survey

^*^Significantly different from zero at the .10 level after adjusting for multiple comparisons, two-tailed test

^**^Significantly different from zero at the .05 level after adjusting for multiple comparisons, two-tailed test

^***^Significantly different from zero at the .01 level after adjusting for multiple comparisons, two-tailed test

**Table 6:** Two-stage least squares impacts of program participation on program components (“Treatment on the Treated”), instrumenting for participation using treatment status and controlling for baseline characteristics (percent, unless otherwise noted)

Program component	Treatment mean	Control mean	Impact	p-Value	Adjusted p-value	Sample size
In previous 12 months, exposed to information on						
Relationships	17.82	20.84	− 3.02	0.680	1.000	221
Parenting	68.24	44.60	23.65	0.010	0.043	220
Child health care	57.28	52.63	4.65	0.614	0.999	221
Education related services	29.44	28.80	0.64	0.930	1.000	220
Career Counseling or job training	18.54	22.05	− 3.51	0.624	0.976	220
Methods of birth control	84.70	67.22	17.48	0.024	0.114	217
Percent correct on assessments of knowledge of contraception						
Condoms	58.55	61.09	− 2.54	0.470	0.920	220
Birth control pills	45.08	55.14	− 10.06	0.050	0.144	220
IUDs	35.38	34.87	0.51	0.904	1.000	220
Other methods	37.11	36.70	0.41	0.923	1.000	220

Treatment and control group means are regression adjusted. Impacts on binary outcomes are estimated using a two-stage least squares model instrumenting for program participation using treatment status, with standard errors adjusted to account for heteroskedasticity. Regressions control for all baseline characteristics and stratum in addition to treatment status. All p-values are based on a two-sided test, and adjusted p-values control for the familywise error rate using the method in Hothorn et al. (2008). Sample sizes differ across outcomes due to missing outcome data. Source: Baseline survey and 12 month follow-up survey

^*^Significantly different from zero at the .10 level after adjusting for multiple comparisons, two-tailed test

^**^Significantly different from zero at the .05 level after adjusting for multiple comparisons, two-tailed test

^***^Significantly different from zero at the .01 level after adjusting for multiple comparisons, two-tailed test

**Table 7:** Impacts on short-term outcomes, excluding controls for baseline characteristics but including controls for randomization strata (percent, unless otherwise noted)

Program component	Treatment mean	Control mean	Impact	p-Value	Adjusted p-value	Sample size
Contraception use in previous 12 months						
Use of a LARC	38.02	28.52	9.50	0.138	0.245	219
Unprotected sex	24.51	25.96	− 1.45	0.806	0.960	218
Respondent intends to wait two or more years before having next child	89.13	94.35	− 5.23	0.16	a	216
Frequency of parental engagement in last month—scale from 0 (never) to 3 (every or almost every day)						
Mother’s engagement with child	2.48	2.48	0.00	0.991	1.000	217
Father’s engagement with child	1.48	1.54	− 0.07	0.651	0.988	211
Quality of co-parenting relationship—scale from 1 to 5 with higher values representing stronger co-parenting	3.74	3.80	− 0.06	0.622	0.983	216
Father pays half or more of child care costs	67.38	69.7	− 2.32	0.715	0.995	216
Capacity for self-sufficiency—scale from 1 to 4 with higher values representing greater self sufficiency	2.15	2.13	0.01	0.688	0.993	221
Child health and development						
Number of well visits	6.34	6.51	− 0.17	0.729	0.926	200
Has health insurance for child	94.65	94.23	0.42	0.895	0.989	216

Treatment and control group means are regression adjusted. Impacts on binary outcomes are estimated using the linear probability model, with standard errors adjusted to account for heteroskedasticity. Regressions control only for treatment status and randomization stratum. All p-values are based on a two-sided test, and adjusted p-values control for the familywise error rate using the method in Hothorn et al. (2008). Sample sizes differ across outcomes due to missing outcome data. Source: Baseline survey and 12 month follow-up survey

^*^Significantly different from zero at the .10 level after adjusting for multiple comparisons, two-tailed test

^**^Significantly different from zero at the .05 level after adjusting for multiple comparisons, two-tailed test

^***^Significantly different from zero at the .01 level after adjusting for multiple comparisons, two-tailed test

^a^In this table, p-values are adjusted for multiple comparisons within outcome domains. Birth spacing intention is the only outcome in its domain, so the adjusted p-value is excluded here

**Table 8:** Two-stage least squares impacts of program participation on short-term outcomes (“Treatment on the Treated”), instrumenting for participation using treatment status and controlling for baseline characteristics (percent, unless otherwise noted)

Program component	Treatment mean	Control mean	Impact	p-Value	Adjusted p-value	Sample size
Contraception use in previous 12 months						
Use of a LARC	40.24	27.63	12.61	0.134	0.239	219
Unprotected sex	23.13	26.95	− 3.82	0.649	0.840	218
Respondent intends to wait two or more years before having next child	89.63	93.39	− 3.75	0.478	a	216
Frequency of parental engagement in last month—scale from 0 (never) to 3 (every or almost every day)						
Mother’s engagement with child	2.48	2.47	0.01	0.907	0.999	217
Father’s engagement with child	1.47	1.54	− 0.08	0.700	0.993	211
Quality of co-parenting relationship—scale from 1 to 5 with higher values representing stronger co-parenting	3.71	3.82	− 0.10	0.479	0.882	216
Father pays half or more of child care costs	66.65	70.10	− 3.45	0.666	0.987	216
Capacity for self-sufficiency—scale from 1 to 4 with higher values representing greater self sufficiency	2.15	2.13	0.02	0.730	0.997	221
Child health and development						
Number of well visits	6.15	6.64	− 0.49	0.439	0.745	200
Has health insurance for child	96.12	93.03	3.09	0.391	0.895	216

Treatment and control group means are regression adjusted. Impacts on binary outcomes are estimated using a two-stage least squares model instrumenting for program participation using treatment status, with standard errors adjusted to account for heteroskedasticity. Regressions control for all baseline characteristics and stratum in addition to treatment status. All p-values are based on a two-sided test, and adjusted p-values control for the familywise error rate using the method in Hothorn et al. (2008). Sample sizes differ across outcomes due to missing outcome data. Source: Baseline survey and 12 month follow-up survey

^*^Significantly different from zero at the .10 level after adjusting for multiple comparisons, two-tailed test

^**^Significantly different from zero at the .05 level after adjusting for multiple comparisons, two-tailed test

^***^Significantly different from zero at the .01 level after adjusting for multiple comparisons, two-tailed test

^a^In this table, p-values are adjusted for multiple comparisons within outcome domains. Birth spacing intention is the only outcome in its domain, so the adjusted p-value is excluded here

## Footnotes

1 The control group did not receive any direct services through the study. Although both the treatment and control groups received information about Project Milestone, the study team was unable to gather data from the WIC clinics about their participation. Similarly, although the HHD also offered home visit services through Nurse Family Partnerships program, young mothers enrolled in that program were not eligible for participation in the study. However, the study team was unable to track whether any teen mothers enrolled in Nurse Family Partnerships at a future date.

2 Because of the small number of teen mothers that the HHD was able to recruit and their early withdrawal from the study study funders did not support a second follow-up at 24 months, when the program would have ended.

3 The number of interview respondents was guided by the available budget. We selected a sample of possible respondents that represented a range of participation in the program and case managers. Nearly all sampled respondents agreed to the interview request; for those that did not, we had a replacement identified that maintained the diversity of the sample.

4 Greater description of the construction of the measures will be included in a forthcoming report to be released by the *Office of Population Affairs*:[author]. “Enhancing home visiting for teen mothers to prevent a rapid repeat birth: An evaluation of Healthy Families Healthy Futures in Houston”. Washington, DC: Office of Populations Affairs, Office of the Assistant Secretary for Health, U.S., Department of Health and Human Services, forthcoming.

5 Some Bayesian statisticians define the prior distribution in terms of beliefs (de Finetti 1974; Kaplan 2019). We define the prior in terms of evidence (Gelman 2015; Deke and Finucane 2019).

6 A meta-analysis of prior evidence requires both impact estimates and some measure of the precision of those estimates (sample size, standard errors, or p-values). HomVEE reports impact estimates and p-values.

7 Our meta-analysis yielded a prior distribution that is Gaussian (normal) with a mean effect size of 0.01 and a standard deviation of 0.10. The meta-analysis included two statistical adjustments to calculate the mean and standard deviation of prior effects. First, we gave greater weight to more precise estimates, a standard practice in meta-analysis (Cooper et al. 2009). Second, to adjust for potential bias due to researchers conducting many analyses but only reporting the most favorable (a phenomenon sometimes called the file drawer problem), we ran a meta-regression of effect size estimates on the standard error of those estimates. This adjustment is motivated by the idea that for any given study, the effect estimate observed in the literature is the largest of all effect estimates calculated by the author (with the rest unseen in a file drawer). In other words, it is a maximum order statistic, which is well approximated by a linear function of the standard error (Royston 1982). The constant term from this meta-regression is our estimate of the mean of the prior distribution.

8 A more robust description of program implementation appears in a report to be released by the *Office of Population Affairs*: [author]. “Enhancing home visiting for teen mothers to prevent a rapid repeat birth: An evaluation of Healthy Families Healthy Futures in Houston”. Washington, DC: Office of Populations Affairs, Office of the Assistant Secretary for Health, U.S., Department of Health and Human Services, forthcoming.

9 The HFHF model was based on HFA; however, the Houston Health Department was not an affiliated site. Only one of the numerous coaches and administrators had attended HFA training before the program began, but that coach was not operating as the recruitment and outreach specialist and was not seeing cases. A small number of staff members attended HFA training during the first year of the study, but doing so took time away from seeing the sample of teen mothers.

10 The probability of an unfavorable effect is one minus the probability of a favorable effect.

11 To test the robustness of these results, we re-ran all our program impact analyses without controls for baseline covariates. We also estimated the impact of actually receiving the HFHF intervention using a two-stage least squares framework. In this approach, we used treatment status as an instrument for the likelihood that an individual attended at least one HFHF visit. Neither approach, whose results are shown in Appendix Tables 5 and 6, and 7 and 8, respectively, yielded qualitatively different outcomes from the main analysis.
