# Supplementary material for: Impacts of a Home Visiting Program Enhanced with Content on Healthy Birth Spacing
Source: Matern Child Health J. 2020 Jul 7;24(Suppl 2):105–18. doi: 10.1007/s10995-020-02968-6 (PMC7497386; doi:10.1007/s10995-020-02968-6)
Supplement: Supplementary file 1 — Supplementary file1 (DOCX 32 kb) [file 10995_2020_2968_MOESM1_ESM.docx]

| Measure | Definition | Source |
| --- | --- | --- |
| **Exposure to information** | |  |
| Exposure to information on relationships | In the past 12 months, did you attend any classes or sessions (individual or group) about the following?  Relationships, dating, or marriage | Adapted from The National Evaluation of the Personal Responsibility Education Program, conducted by Mathematica under contract with the Administration for Children and Families (ACF) |
| Exposure to information on parenting | In the past 12 months, did you attend any classes or sessions (individual or group) about the following?  Parenting or how to care for your child? | Adapted from The National Evaluation of the Personal Responsibility Education Program, conducted by Mathematica under contract with the Administration for Children and Families (ACF) |
| Exposure to information on child health care | In the past 12 months, did you attend any classes or sessions (individual or group) about the following?  How to get insurance or apply for Medicaid for their child  Where to get health care for their child | New item developed by Mathematica for this evaluation |
| Exposure to education-related services | In the past 12 months, have you participated in any of the following education related services?  GED preparation, tutoring, or outside help with school work, or program to prepare for a high school diploma | Adapted from the Evaluation of the Impact of Youth Build, conducted by Mathematica under contract with |
| Exposure to career counseling or job training | In the past 12 months, have you participated in any of the following training or job related services?  Career counseling or job training | Adapted from the Evaluation of the Impact of the Youth Build Program Follow-Up Survey-used with an adolescent/young adult population |
| Exposure to information about methods of birth control | In the past 12 months, did you receive information from a doctor, nurse, home visitor, or clinic about any of the following?  Methods of birth control, such as condoms, birth control pills, the patch, the shot, the ring, IUD, or an implant | Adapted from The National Evaluation of the Personal Responsibility Education Program, conducted by Mathematica under contract with the Administration for Children and Families (ACF) |
| **Knowledge of contraception** | |  |
| Knowledge of condoms | Please mark whether you think each statement is true, false, or you don’t know.   - It is okay to use the same condom more than once. - Condoms have an expiration date. - When putting on a condom, it is important to leave space at the tip. - It is ok to use petroleum jelly or Vaseline as a lubricant when using latex condoms. - When using a condom, it is important for the man to pull out right after ejaculation. - Wearing two latex condoms will provide extra protection. | Fog Zone/Power to Decide, a survey designed for unmarried young adults regarding pregnancy planning, contraception,  and related issues. They developed items based on other national surveys. |
| Knowledge of birth control pills | Please mark whether you think each statement is true, false, or you don’t know.   - Birth control pills are effective, even if a woman misses taking them for two or three days in a row. - Women should “take a break” from the pills every couple of years. - After a woman stops taking birth control pills, she is unable to get pregnant for at least two months. - In order to get the birth control pill, a woman must have a pelvic exam.   Birth control pills can reduce risk of getting a sexually transmitted disease or STD. |  |
| Knowledge of IUDs | Please mark whether you think each statement is true, false, or you don’t know.   - Women who use IUDs cannot use tampons. - A woman can get an IUD without going to a doctor’s office, clinic, or medical professional. - An IUD cannot be felt by a woman’s partner during sex. - IUDs can move around in a woman’s body. - An IUD is effective (prevents pregnancy) for at least 3 years. - Using an IUD will cause weight gain. |  |
| Knowledge of other hormonal and LARC methods | Please mark whether you think each statement is true, false, or you don’t know.   - Women using the birth control shot, Depo-Provera, must get an injection about every 3 months. - Women using the vaginal ring, or NuvaRing, must have it inserted by a doctor or health care provider every month. - Long-acting methods like the implant (Implanon or Nexplanon) or an IUD (Mirena, ParaGard, or Skyla) cannot be removed early, even if a woman changes her mind about wanting to get pregnant. - Long-acting methods like the implant (Implanon or Nexplanon) or an IUD (Mirena, ParaGard, or Skyla) can make it more difficult to become pregnant in the future when a woman is no longer using them. |  |
| **Contraception use** | |  |
| Use of a LARC | Are you currently using …  a. Condoms?  b. Birth control pills?  c. The patch, such as Ortho Evra?  d. The shot, such as Depo-Provera or other injectable birth control?  e. The ring, such as NuvaRing?  f. An IUD, such as Mirena, ParaGard, or Skyla?  g. An implant, such as Implanon or Nexplanon?  h. Another type of birth control? | Adapted from The National Evaluation of the Personal Responsibility Education Program, conducted by Mathematica under contract with the Administration for Children and Families (ACF) |
| Unprotected sex | In the past 3 months, have you had sexual intercourse? By sexual intercourse we mean a male putting his penis into a female’s vagina.  In the past 3 months, did you ever have sexual intercourse without using birth control, such as condoms, birth control pills, the patch, the shot, the ring, an IUD, or an implant? | Adapted from The National Evaluation of the Personal Responsibility Education Program, conducted by Mathematica under contract with the Administration for Children and Families (ACF) |
| **Healthy birth spacing** | |  |
| Intention to space births | How soon would you like to have your next child? Would you like to have it…  Within the next year,  One year from now,  Two years from now,  Three years from now,  Four or more years from now? | Adapted from The National Evaluation of the Personal Responsibility Education Program, conducted by Mathematica under contract with the Administration for Children and Families (ACF) |
| **Enhanced family functioning** | |  |
| Mother’s engagement in play with child | **In the past month, how often have you done the following activities (more than once a day, every day or almost every day, a few times a week, a few times in the past month, once or twice in the past month, or never):**   - Played games like “peek-a-boo” or “gotcha” with child. - Sung songs with child. - Read or looked at books with child. - Played outside or at the playground with child. - Played with games or toys with child. | Adapted from the National Evaluation of Building Strong Families, conducted by Mathematica under contract with ACF. Items originated on the survey for the Fragile Families Longitudinal Study. |
| Father’s engagement with child | **In the past month, how often has your child’s father done the following activities (more than once a day, every day or almost every day, a few times a week, a few times in the past month, once or twice in the past month, or never):**   - Played games like “peek-a-boo” or “gotcha” with child. - Sung songs with child. - Read or looked at books with child. - Played outside or at the playground with child. - Played with games or toys with child. - Helped child to get dressed. - Changed child’s diapers or helped him/her use the toilet. - Given child a bottle or something to eat. | Adapted from the National Evaluation of Building Strong Families, conducted by Mathematica under contract with ACF. Items originated on the survey for the Fragile Families Longitudinal Study. |
| Quality of co-parenting relationship | **For each statement, please answer if you strongly agree, agree, are not sure, disagree, or strongly disagree.**   - I feel good about child’s father’s judgement about what is right for child. - Child’s father and I are a good team (5.30b). - When there is a problem with child, child’s father and I work out a good solution together (5.30c). - Child’s father makes my job of being a parent easier. - Child’s father pays a great deal of attention to child. - Child needs child’s father just as much as he needs me - No matter what might happen between child’s father and me, when I think of child’s future, it includes child’s father | Adapted from the National Evaluation of Building Strong Families, conducted by Mathematica under contract with ACF. Items originated on the survey for the Fragile Families Longitudinal Study. Also used by The National Evaluation of the Personal Responsibility Education Program and the Evaluation of Parents and Children Together, conducted by Mathematica for ACF. |
| Financial support from father | Parents deal with meeting the expenses of raising a child in different ways. When answering the next question, I’d like you to think about all the expenses associated with raising [CHILD] such as [CHILD]’s food, clothing, medical expenses, diapers, and any other costs of raising [CHILD].  How much of the cost of raising [CHILD] does [CHILD]’s father cover? Would you say it’s…    All or almost all,  More than half,  About half,  Less than half  Little or none? | Adapted from the National Evaluation of Building Strong Families, conducted by Mathematica under contract with ACF. Items originated on the survey for the Fragile Families Longitudinal Study. Also used by The National Evaluation of the Personal Responsibility Education Program |
| Capacity for self-sufficiency— Goals, plans, problem solving | **How much do you agree or disagree with the following statements?**   - I am focused on preventing negative things from happening in my life. - I set goals and think about what I need to do to reach those goals. - When faced with a problem, I can usually find a solution. - I think going to college is important for getting a good job. - I am focused on achieving good and positive things in my life. - I have a plan for achieving my future education or career goals. - I don’t like to plan too far ahead because things don’t usually go the way I planned | Developed by the California Department of Public Health, Division of Maternal and Child Health for their evaluation of their state-wide program for pregnant and parenting teens. |
| **Child health and development** | |  |
| Number of well visits | During the past 12 months, how many times did [CHILD] see a doctor, nurse, or other health care provider for a regular checkup, not a sick child care visit or hospitalization? Your best estimate is fine.  Yes  No | New item developed by Mathematica for this evaluation |
| Health insurance for child | The next questions are about health insurance. This can include private insurance, Medicaid/STAR, or any other government program that pays for medical care.  Do you have health insurance for [CHILD]?  Yes  No | New item developed by Mathematica for this evaluation |
